# Obituary

**Published:** 1886-05

**Authors:** 


### Obituary.

Died at his residence in Newport, Ky., on the 27th inst., in his 74th year, Dr. J. M. Brown, M.D.

Dr. Brown was an old resident of Cincinnati, he was a graduate of medicine, and in his earlier life practiced his profession in Hillsboro, Ohio, and was very successful. His field of practice was too laborious, and about 1847, he came to Cincinnati and opened the first dental depot, west of the Allegheny Mountains, in connection with which he opened a drug store, His business place was in the corner room of the Melodeon Building, corner of Fourth and Walnut streets.

His going into dental supplies was regarded by many at that time as a great venture, and one that could not at best give good business returns. He was encouraged to enter upon and continue in the business by the leading dentists of this city.

He persevered till the dental depot was a complete business success.

He continued in this building for about thirty years ; during this time he engaged in several other enterprises, he was for many years a member of the banking house of Fallis, Brown & Co., also a member of the firm of Adams, Peckover & Co., stove manufacturers. He was interested from time to time in several other enterprises.

Dr. Brown was an enterprising and public-spirited citizen, he possessed a sympathetic and warm-hearted nature, genial and sociable to all.

The older members of the dental profession will with special sadness learn of his death. He leaves an estimable wife, who will have the warm sympathy of all the Doctor's friends and acquaintances.

